# Wernicke's encephalopathy with chorea: Neuroimaging findings

**DOI:** 10.1590/s1980-5764-2016dn1004020

**Published:** 2016

**Authors:** Jivago S. Sabatini, Gustavo Leopold Schutz-Pereira, Fabrício Feltrin, Hélio Afonso Ghizone Teive, Carlos Henrique Ferreira Camargo

**Affiliations:** 1 MD. Neurology Service, Hospital Universitário, State University of Ponta Grossa, Paraná, Brazil.; 2 MD. Neurology Service, Hospital Universitário, State University of Ponta Grossa, Paraná, Brazil.; 3 MD. Radiology Unit, Hospital Universitário, State University of Ponta Grossa, Paraná, Brazil.; 4 MD, PhD. Movement Disorders Unit, Neurology Service, Hospital de Clínicas, Federal University of Parana, Curitiba - Paraná, Brazil.; 5 MD, PhD. Neurology Service, Hospital Universitário, State University of Ponta Grossa, Paraná, Brazil.

**Keywords:** Wernicke's encephalopathy, thiamine deficiency, dementia, chorea, movement disorders

## Abstract

We present a case report of motor and cognitive disorders in a 36-year-old woman with a history of twelve years of heavy alcohol abuse. The patient presented depressive symptoms over the course of one year after a loss in the family, evolving with ataxia, bradykinesia and choreiform movements. Progressive cognitive decline, sleep alterations and myalgia were also reported during the course of disease evolution. Physical examination revealed spastic paraparesis with fixed flexion of the hips and knees with important pain upon extension of these joints. Initial investigation suggested the diagnosis of thiamine deficiency by brain magnetic resonance imaging (MRI).

## Abstract (pt)

Um relato de caso de transtornos motor e cognitivo em uma mulher de 36 anos, com história de doze anos de abuso de álcool. A paciente apresentou sintomas depressivos ao longo de um ano após uma perda na família, evoluindo com ataxia, bradicinesia e movimentos coreiformes. O declínio cognitivo progressivo, alterações do sono e mialgia também foram relatados durante o curso da evolução da doença. O exame físico demonstrou paraparesia espástica com flexão fixa dos quadris e joelhos com dor importante na extensão dessas articulações. A investigação inicial sugeriu o diagnóstico de deficiência de tiamina por ressonância magnética cerebral (MRI).

## INTRODUCTION

**Figure 1:**
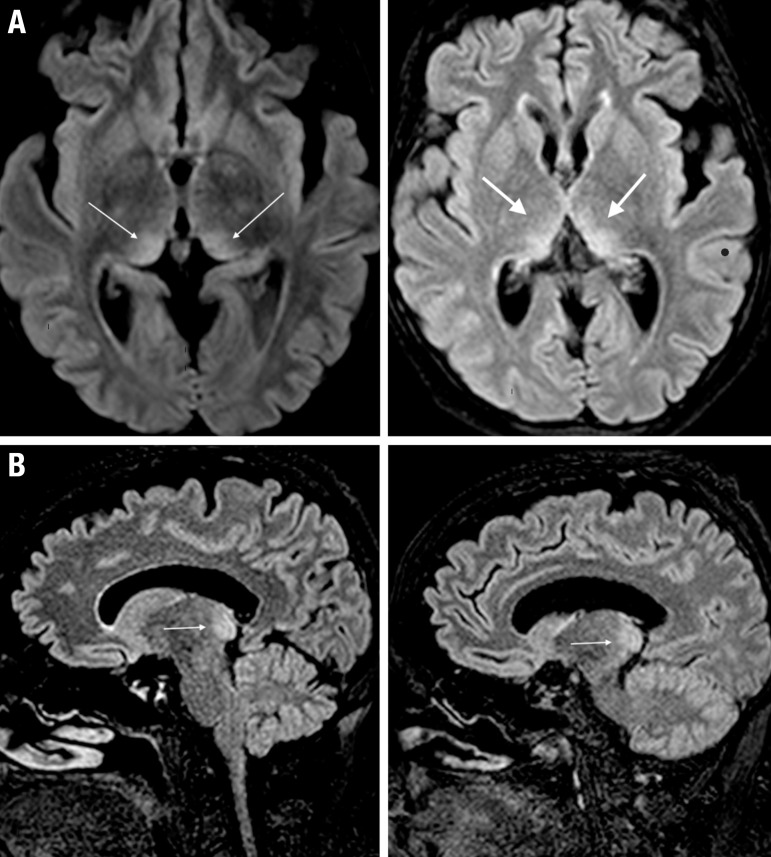
[A] Axial FLAIR-weighted MRI images showing signal hyperintensity in the medial and pulvinar regions of both thalami. [B] Sagittal FLAIR-weighted MRI images showing signal hyperintensity in the pulvinar of both thalami.

**Figure 2:**
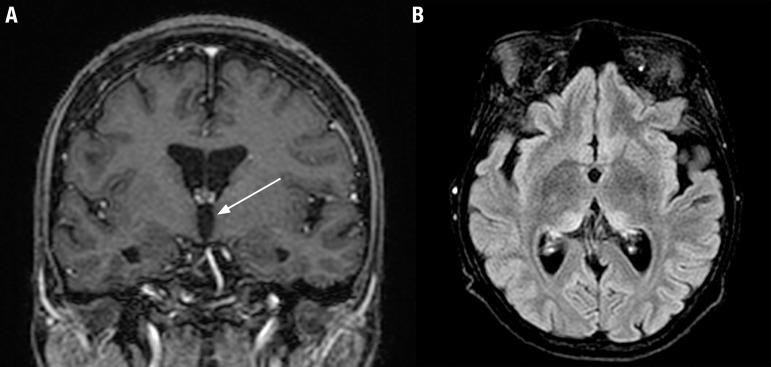
[A] Coronal T1-weighted postcontrast magnetic resonance image reveals no abnormal enhancement of the mammillary bodies (arrows). [B] Axial fluid-attenuated inversion recovery (FLAIR) image reveals abnormal signal at the periventricular region of both thalamus that persisted after treatment.

This case report describes motor and cognitive disorders in a 36-year-old woman with a history of 12 years of alcohol abuse. The patient presented weight loss and depressive symptoms over the course of one year after a loss in the family, evolving with ataxia, nystagmus, bradykinesia, vertigo, choreiform movements of the upper limbs and spastic paraparesis eight months after disease onset. Progressive cognitive decline, sleep alterations and myalgia were also reported during disease evolution. The physical examination revealed spastic paraparesis (fixed flexion of hips and knees, with important pain upon extension of these joints), hyporeflexia and choreic movements in distal arms. Accurate and complete cognitive assessment was difficult due to intense agitation and aggression, as well as speech and language impairments. Initial investigation by brain magnetic resonance imaging (MRI) showed signal hyperintensity in pulvinar thalami (Figure 1). The electroencephalogram (EEG) showed a disorganized pattern with bursts of intermittent slow waves. An electroneuromyography study performed during the hospital stay disclosed severe motor and sensorial axonal polyneuropathy, with signs of ongoing denervation. Unfortunately, serum thiamine measurement was not available. However, taking into account the history of alcohol abuse, the clinical and MRI findings, and the presence of peripheral neuropathy compatible with a dry beriberi pattern, it was decided to administer prompt thiamine replacement. Soon after starting therapy, the patient presented with remarkable regression of motor and cognitive symptoms, including the disappearance of choreiform movements. The patient had a Mini-Mental State Examination (MMSE) score of 23/30 at discharge after 2 weeks, and a score of 28/30 six months after hospitalization. Spastic paresis of the lower limbs persisted, later treated with local injections of botulinum toxin. Despite the improvement of clinical features, five months after discharge a new MRI study showed persistence of hyperintensity on T2 and FLAIR sequences in both medial thalami and pulvinar nuclei. No classical mammillary body hyperintensity was evident after gadolinium injection on the two exams (Figure 2).

Wernicke's encephalopathy (WE) is a clinical syndrome that results from thiamine (vitamin B1) deficiency. The clinical findings that characterize the syndrome are nystagmus, ophthalmoplegia, mental status changes and cerebellar dysfunction. Uncommon manifestations of the disease at presentation include epileptic seizures, stupor, hypotension, tachycardia, visual disturbances, hearing loss and hallucinations. In later stages, patients may present with choreic dyskinesias, increased muscular tone and spastic paresis, hyperthermia and even coma. MRI is currently considered the best method for confirming diagnosis of this condition and typically shows a bilateral symmetric hypersignal in the paraventricular thalamic nuclei on T2-weighted images.^1,2,3^ Other less frequent sites of signal alterations include the mammillary bodies, the tectal plate and, more frequently, the periaqueductal area.^6^ Although thalamic hyperintense signal may be found in other diseases (Creutzfeldt-Jakob disease, Fabry's disease, thalamic infarction), the clinical course described in this case strongly suggested thiamine deficiency. Some studies have reported reversion of thalamic hyperintensity after treatment, but this has not occurred in our case to date.^9^ EEG may show non-specific slowing of the dominant rhythm at a late stage, proving important in this case to exclude characteristic changes of Creutzfeldt-Jakob disease.^4^

Response to thiamine replacement is usually satisfactory, with resolution of ocular symptoms within hours, motor symptoms in days and mental status improvement over the course of weeks. It should be noted that inappropriate treatment or unrecognized WE may evolve to Korsakoff syndrome (KS), resulting in lasting cognitive symptoms, such as anterograde amnesia.^8^ "Dry" beriberi is a peripheral manifestation of thiamine deficiency, usually presenting as an axonal motor and sensory polyneuropathy. The symptoms resolve comparatively more slowly than the symptoms of WE, taking from 3 to 6 months to improve after initial thiamine replacement.^7,10,11^ There is no consensus with regard to the optimal dose of the therapy, however, it is well established that the disease should be treated using intravenous or intramuscular injections immediately after diagnosis to ensure adequate absorption.

This case suggests that faster diagnosis with clinical and MRI features of WE can allow rapid thiamine replacement with good response of severe symptoms such as choreiform movements.
